# Parametrically Amplified Bright-state Polariton of Four- and Six-wave Mixing in an Optical Ring Cavity

**DOI:** 10.1038/srep03619

**Published:** 2014-01-09

**Authors:** Haixia Chen, Yiqi Zhang, Xin Yao, Zhenkun Wu, Xun Zhang, Yanpeng Zhang, Min Xiao

**Affiliations:** 1 Key Laboratory for Physical Electronics and Devices of the Ministry of Education & Shaanxi Key Lab of Information Photonic Technique, Xi'an Jiaotong University, Xi'an 710049, China; 2 Department of Physics, University of Arkansas, Fayetteville, Arkansas 72701, USA & National Laboratory of Solid State Microstructures and Department of Physics, Nanjing University, Nanjing 210093, China

## Abstract

We report experimental studies of bright-state polaritons of four-wave mixing (FWM) and six-wave mixing (SWM) signals through cascade nonlinear optical parametric amplification processes in an atom-cavity composite system for the first time. Also, the coexisting cavity transmission modes of parametrically amplified FWM and SWM signals are observed. Finally, electromagnetically induced absorption by the FWM cavity modes in the probe beam is investigated. The investigations can find potential applications in multi-channel narrow-band long-distance quantum communication.

Cavity quantum electrodynamics has been intensively studied in recent years^1^,^2^,^3^,^4^, owing to the important applications in quantum optics and nonlinear optics. When a two-level atom is placed inside the optical cavity under the strong coupling condition, the “normal-mode splitting” have been experimentally demonstrated in many atomic systems. If the intracavity medium is a coherently-prepared three-level atomic medium, the so called “dark-state polariton” peak^5^,^6^,^7^ appears in the cavity transmission spectrum (CTS) due to the intracavity electromagnetically induced transparency (EIT). Such “dark-state polariton” have potential application in the long-lived storage of quantum information and quantum computation. Moreover, when the atomic system is in the double-Λ configuration, correlated photon pairs with high generation rate and narrow bandwidth have been generated^8^ in the cold atomic ensemble by using an optical parametric amplification (OPA) process operated below its oscillation threshold. Meanwhile, bright correlated anti-Stokes and Stokes twin beams with hot atoms inside an optical cavity have also been obtained by an OPA process above the threshold^9^. When an ensemble of cold atoms strongly coupled to a high-finesse optical cavity, and the control light can be generated from the vacuum, then the vacuum-induced transparency was observed^10^. In addition, four-wave mixing (FWM) and six-wave mixing (SWM) processes based on third-order and fifth-order nonlinear processes in EIT media^11^,^12^,^13^ have also attracted lots of attention in recent years, in which a strong coupling beam renders a resonant, opaque medium nearly transparency while enhancing the nonlinearity, and the coexistence of these two nonlinear processes due to double EIT windows and atomic coherences has been reported^14^,^15^. The nonlinear process plays important role in entanglement generation^16^,^17^ and cascade-nonlinear optical process^18^. To the best of our knowledge, the OPA seeded with FWM or SWM signal in an atom-cavity composite system has not been reported, and the investigation of it would be important for building multi-channel nonlinear optical devices and ultra-narrow linewidth photon sources for long-distance quantum communication^19^.

In this letter, we report our investigations of bright-state polaritons of FWM and SWM signals through cascade OPA process in an atom-cavity composite system. The bright-state polaritons of FWM signal with 5 MHz linewidth are obtained. We also report the coexisting cavity modes of parametrically amplified FWM and SWM signals in an inverted-Y-type atomic system for the first time. Moreover, the electromagnetically induced absorption (EIA) peaks induced by the multiple cavity modes of the FWM signal are observed in the probe beam. These results are well explained by the presented theoretical model. The investigation will help us to better understand the interactions between the strongly coupled multi-level atoms and the optical cavity, which can find application in quantum information processing.

## Results

**Figure 1:**
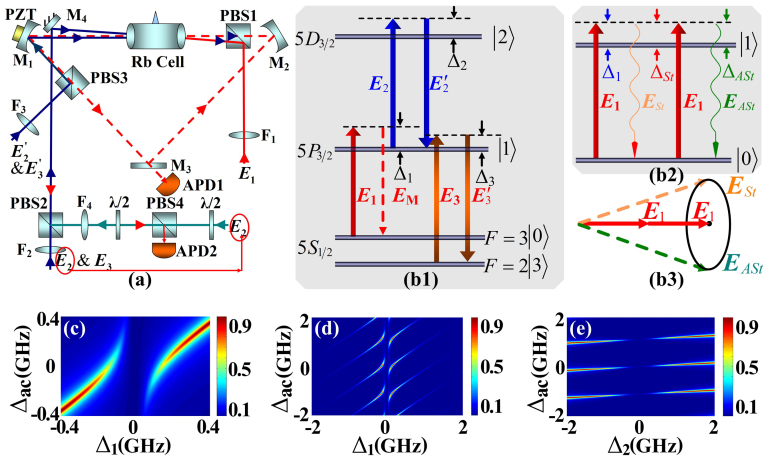
(a) Experimental setup. PBS: polarizing beam splitters; F: optical lens; APD: avalanche photodiode detectors; PZT: piezoelectric transducer; M1–M3: cavity mirrors; M4, high reflectivity mirror; *λ*/2: half-wave plate. (b1) Energy levels for the ^85^Rb atomic system. (b2) Energy schematic of SP-FWM process. (b3) phase-matching geometrical diagram of SP-FWM process. Calculated CTS versus Δ_1_ and Δ*_ac_* by using the expression of *a_F_*_1_ (c) in a FSR and (d) in multiple FSRs. (e) Calculated CTS versus Δ_2_ and Δ*_ac_* by using the expression of *a_F_*_1_ in multiple FSRs.

Fig. 1(a) shows the experimental setup, while the energy levels of the atomic system used in the experiment is shown in Fig. 1(b1), where the fields ***E***_1_ (frequency *ω*_1_, wave vector ***k***_1_, Rabi frequency *G*_1_, and wavelength 780.2 nm), ***E***_2_ (*ω*_2_, ***k***_2_, *G*_2_, and 776.16 nm) & 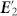 (*ω*_2_, 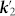, 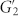), and ***E***_3_ (*ω*_3_, ***k***_3_, *G*_3_, and 780.2 nm) & 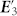 (*ω*_3_, 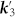, 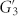) are used as probe field, pumping fields and coupling fields, respectively. The resonant transition frequencies of |0〉→|1〉, |1〉→|2〉 and |1〉→|3〉 are Ω_1_, Ω_2_ and Ω_3_, respectively. Then the frequency detuning for each field can be defined as Δ*_i_* = Ω*_i_* − *ω_i_* (*i* = 1, 2, 3). With all beams present in Fig. 1(b1), three phase-conjugate signals (FWM signals ***E****_F_*_1_ & ***E****_F_*_2_ and SWM signal ***E****_S_*), satisfying the phase-matching conditions of 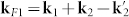, 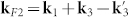, and 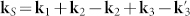, can be generated simultaneously at the center of the atomic cell and propagate along the optical axis of the cavity (dashed line in Fig. 1(a)). Here, the narrow signals ***E****_F_*_1_ and ***E****_S_* are generated within the EIA window of δ = 0, where δ = Δ_1_ + Δ_2_ is two-photon detuning, while the broad signal ***E****_F_*_2_ will be generated due to no assistance of Doppler-free atomic coherence and EIA window. However, if the ***E***_1_ beam has a sufficiently high power, as well as is far detuned from |0〉→|1〉, a spontaneous parametric FWM (SP-FWM) process will occur in the degenerate two-level atomic configuration (Fig. 1(b2)), which generates two weak fields (Stokes field ***E****_St_* and anti-Stokes field ***E****_ASt_*), satisfing 2**k**_1_ = **k***_St_* + **k***_ASt_* (Fig. 1(b3)), on a forward cone. The generated ***E****_F_*_1_ (or ***E****_S_*) signal is naturally injected into the input Stokes port of the SP-FWM process, and is parametrically amplified, where the process will serve as an OPA. The parametrically amplified signal denoted as 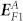 (or 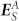) is still generated at the center of the atomic cell and propagate along the optical axis of the cavity, so they are mode-matched to the cavity and form the cavity modes.

According to the expression of *a_F_*_1_ given in the Method part, Fig. 1(c) shows the calculated normalized CTS versus Δ*_ac_* (defined as Δ*_ac_* = Ω_1_ − *ω_c_* with *ω_c_* being the resonant frequency of the cavity) and Δ_1_, which exhibits a double-peak structure along the Δ_1_ direction. This structure indicates the cavity polaritons^7^ of the parametrically amplified field 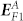, which results from the coupling between the cavity mode of 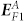 and atoms with strength 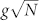, where g is the single-atom-cavity coupling strength, and *N* is the number of atoms in the cavity. When Δ*_ac_* changes from negative to positive continuously, the polaritons move, that is dictated by the condition of Δ_1_ − Δ*_ac_* = 0, and finally leads to the avoided-crossing plot in Fig. 1(c). Fig. 1(d) is plotted with the same variables as Fig. 1(c) but in a wider range, where multiple polaritons at a fixed Δ_1_ can be seen, and their positions change as Δ_1_ changes. It is found that Δ*_F_*_1_ − Δ*_ac_* = *lω_FSR_* (*l* is an integer, and *ω_FSR_* is the free-spectral range (FSR) with medium) is always fulfilled at all the polaritons, i.e. Δ_1_ − Δ*_ac_* = *lω_FSR_* (named cavity transmission window) is satisfied for all the polaritons. So, when Δ_1_ changes, the corresponding Δ*_ac_* for each transmission polaritons of 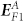 changes accordingly. We also show normalized CTS versus Δ_2_ and Δ*_ac_* in Fig. 1(e), in which the polaritons nearly do not move with Δ_2_. The reason is that the energy shifts induced by dressing field ***E***_2_ at different Δ_2_ values make the window Δ_1_ − Δ*_ac_* = *lω_FSR_* not move.

It is worth mentioning that the cavity polaritons of the parametrically amplified field 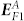 and 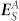 will have ultra-narrow linewidths, which would be useful for long-distance quantum communications. On the one hand, the linewidths of 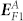 and 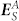 are narrowed by the EIA window, on the other hand, 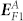 and 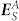 form cavity modes, and then the cavity polaritons will be further narrowed due to the large dispersion change and reduced absorption accompanying EIA^20^,^21^.

### Parametrically amplified bright-state polariton of FWM signal

**Figure 2:**
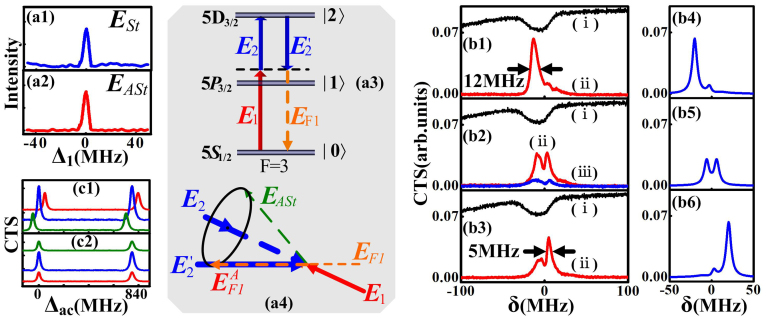
(a1) Measured Stokes field ***E****_St_* and (a2) anti-Stokes field ***E****_ASt_* versus Δ_1_ in the SP-FWM process. (a3) Ladder-type atomic levels to generate ***E****_F_*_1_. (a4) Phase-matching diagram of OPA seeded with the ***E****_F_*_1_ in the Stokes port. (b1)–(b3) Measured probe absorption (i) and CTS (ii) versus δ at different Δ*_ac_*. (b4)–(b6) Theoretical simulation of the curves (ii) in Figs. (b1)–(b3), respectively. (c1) and (c2) Measured polaritons of 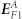 versus Δ*_ac_* with increasing Δ_1_ and Δ_2_ from bottom to top, respectively.

We first measure the CTS without ***E***_3_ and 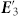. In this case, the SP-FWM process generates a Stokes field ***E****_St_* (measured and shown in Fig. 2(a1)) and an anti-Stokes field ***E****_ASt_* (measured and shown in Fig. 2(a2)) on a forward cone. Such a process can also act as an OPA for the ***E****_F_*_1_ (in Fig. 2(a3)) injected into the Stokes port (in Fig. 2(a4)). The parametrically amplified signal denoted as 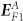 forms cavity mode, which couples with the atoms. By scanning Δ_1_ across the transition *F* = 3↔*F*′ in ^85^Rb at different Δ*_ac_* values and taking Δ_2_ ≈ 1.2 GHz, the measured CTS of 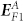 versus δ are shown in Figs. 2(b1)–(b3), where the lower curves (ii) and top curves (i) are the CTS of 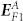 and the corresponding EIA window, respectively, with the atomic cell temperature *T* ≈ 77°C, and powers of ***E***_1_, ***E***_2_, and 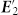 *P*_1_ = 8 mW, *P*_2_ = 22 mW, *P*′_2_ = 22 mW, respectively. The CTS of 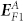 without the atom-cavity mode coupling is shown in Fig. 2(b2) by the curve (iii), where one dip and two peaks can be seen clearly. The two peaks represent the Autler-Townes splitting of the signal 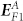, which derive from the two dressed states, namely, bright states. The dip comes from the dark state induced by the destructive interference between two dressed states. Tuning Δ*_ac_* with the atom-cavity mode coupling indicates that, when the Δ_1_ − Δ*_ac_* = 0 window is overlapped with the EIA window, the two peaks reach their maxima simultaneously on the curve (ii) of Fig. 2(b2). Comparing the curve (iii) with the curve (ii), the dip (corresponding to dark state) and the two peaks is amplified, which stems from the dressing splitting of the atom-cavity mode coupling to the energy level |1〉. So the two peaks on the curve (ii) represent the cavity polaritons of 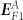 as shown in Fig. 1(c). When the Δ_1_ − Δ*_ac_* = 0 window deviates from EIA window by decreasing Δ*_ac_*, the two peaks become asymmetric, and the left peak is amplified by the atom-cavity mode coupling as shown in Fig. 2(b1). The left peak corresponds to one of the bright states, so one intracavity bright state (“bright-state polariton” with a linewidth about 12 MHz) is obtained. The other narrow “bright-state polariton” (~5 MHz) is obtained when increasing Δ*_ac_* in Fig. 2(b3). The calculated CTS curves of 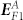 (∝|*a_F_*_1_|^2^), which agree well with the measured results, are shown in Figs. 2(b4)–2(b6).

By scanning the voltage imposed on PZT, Fig. 2(c1) displays the parametrically amplified bright-state polaritons of 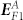 versus Δ*_ac_* at different δ values, which is set as −12 MHz, 0 and 12 MHz from bottom to top by taking different Δ_1_ with fixed Δ_2_. Each CTS curve exhibits two bright polaritons, satisfying Δ_1_ − Δ*_ac_* = *lω_FSR_*, having a separation of *ω_FSR_*, and changing their positions with Δ_1_ as predicted by the theoretical result in Fig. 1(d). Also, the heights of the polaritons change with Δ_1_, since the polaritons are enhanced at two-photon resonant. Next, by fixing Δ_1_ and taking different Δ_2_, the bright-state polaritons of 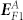 versus Δ*_ac_* with δ set as −15 MHz, 0 and 15 MHz from bottom to top are shown in Fig. 2(c2), where the heights of the polaritons change with Δ_2_ due to two-photon detuning, but the positions are fixed. That is similar to the case shown in Fig. 1(e).

**Figure 3:**
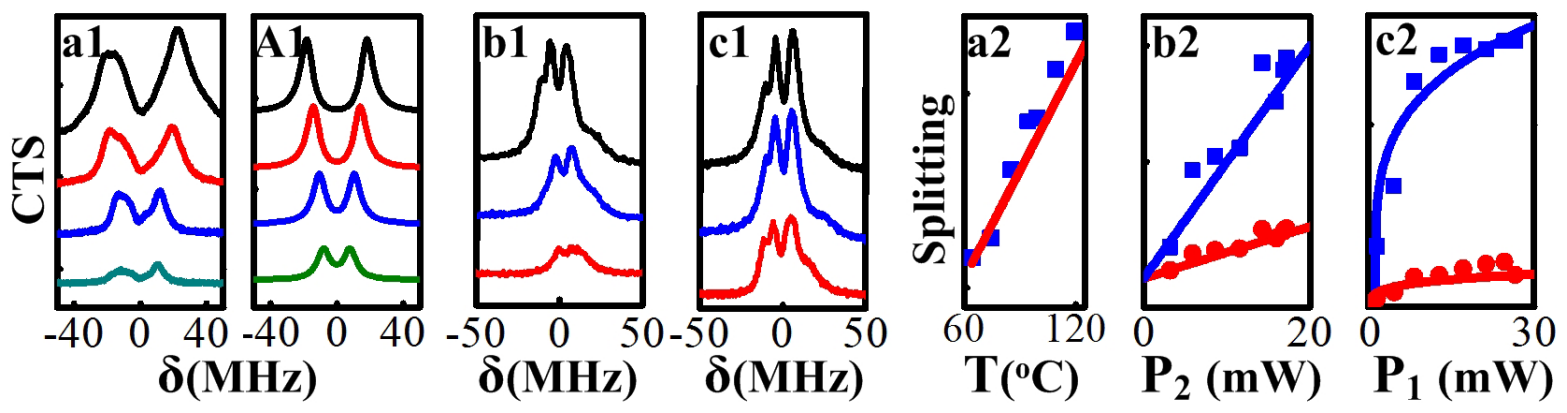
(a1) Measured polaritons of the 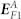 versus δ with Δ_2_ ≈ 1.2 GHz and increasing *T* from bottom to top (65°C, 75°C, 85°C, 95°C). (A1) Theoretical simulation of the curves in (a1). (a2) Measured (squares) and Calculated (solid line) dressing splittings versus *T*. (b1) Measured polaritons with increasing *P*_2_ from bottom to top (3.3 mW, 8.6 mW, 17 mW). (b2) *P*_2_-dependence of the splitting with (top curves) and without (bottom curves) atom-cavity mode coupling. (c1) and (c2) are plotted in the same way as (b1) and (b2) but with increasing *P*_1_ (2.5 mW, 6 mW, 12 mW).

The polaritons of 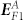 can be affected by the temperature of atomic cell, since increasing *T* can increase the atomic number *N*, and then enlarge the collective coupling factor 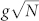
7, which will yield dressing to the polaritons. According to the theoretical model presented in the method part, the splitting eigenvalues induced by the dressing of atom-cavity mode coupling to the polaritons can be given by 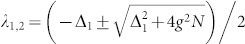, which correspond to the positions of the two polariton peaks in the CTS curves. So the splitting of the two polariton peaks will increase with *T*. Also, increasing temperature will lead to increased atomic number participating in the phase-conjugate FWM process as well as OPA process, which will enhance the height of the two polariton peaks. The measured CTS curves versus δ with increasing *T* from bottom to top are shown in Fig. 3(a1), where not only the height but also the splitting of the two polariton peaks increase with *T*. Fig. 3(A1) shows the calculated results corresponding to Fig. 3(a1), according to the expression of *a_F_*_1_. The experimentally measured (squares) and theoretically calculated (solid line) splittings of polaritons versus *T* are shown in Fig. 3(a2), where the splitting indeed increases with *T*. The experimental results agree with the theory.

The polaritons of 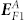 can also be influenced by the powers *P*_1_ and *P*_2_. The measured CTS curves versus δ with increasing *P*_2_ from bottom to top are presented in Fig. 3(b1). The height and the splitting of the two polariton peaks become more pronounced as *P*_2_ increase. Figure 3(b2) shows the splitting versus *P*_2_ with the atom-cavity mode coupling (upper plots) and without the atom-cavity mode coupling (lower plots), where squares and dots are experimental results, while the solid curves are the corresponding fittings. The splitting on upper plots in Fig. 3(b2) is mainly induced by the dressing of the atom-cavity mode coupling and field ***E***_2_ to polaritons, while the splitting on lower plots mainly results from the dressing of the field ***E***_2_. By comparing the two cases, the dressing splitting of the atom-cavity mode coupling can be obtained. While the dressing splitting induced by field ***E***_2_ to the polariton can be seen by increasing *P*_2_. Similar investigation has been done for the influence of *P*_1_ in Figs. 3(c1) and 3(c2). However, the upper squares (or solid line) in Fig. 3(c2) is much easier to saturate than that in Fig. 3(b2). The dressing splitting of the polariton in Fig. 3(c2) is mainly determined by the atom-cavity coupling and field ***E***_2_ when the power of *P*_1_ is weak, which is similar to the case in Fig. 3(b2). As *P*_1_ is increased, the dressing of field ***E***_1_ gets larger and has to be considered. So the dressing splitting of field ***E***_1_ to polaritons is included in Fig. 3(c2) compared with Fig. 3(b2). Therefore, the saturation behavior with increasing *P*_1_ in Fig. 3(c2) mainly results from the balanced interactions between the destructive and constructive interferences of different dressing pathways^22^ induced by the atom-cavity mode coupling, field ***E***_1_ and field ***E***_2_.

### Coexisting cavity modes of parametrically amplified FWM and SWM signals

**Figure 4:**
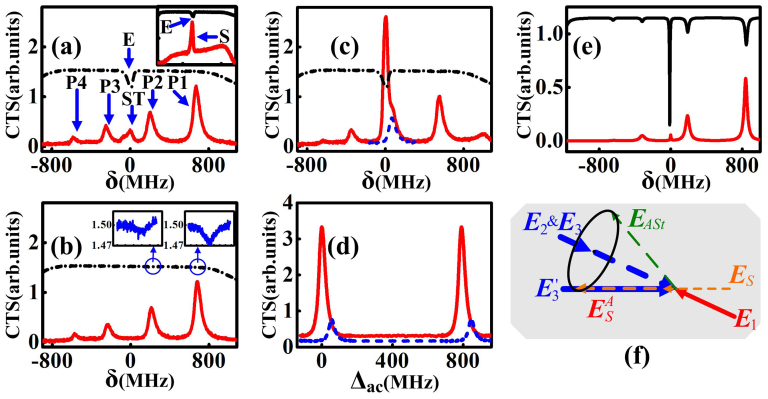
(a)–(c) Measured CTS of ***E****_F_*_2_ and 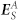 signals versus δ at different Δ*_ac_* values. Coexisting 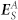 and ***E****_F_*_2_ signals versus δ in free space shown in the inset of (a), and measured FWM-induced EIA dips shown in the insets of (b). (d) Measured CTS of ***E****_F_*_2_ and 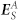 signals versus Δ*_ac_* with (solid curve) and without (dashed curve) ***E***_2._ (e) Calculated FWM-induced EIA (top curve) and CTS (bottom curve) with coexisting ***E****_F_*_2_ and 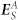 versus δ. (f) Phase-matching of OPA process seeded with ***E****_S_*.

If all laser beams are opened except 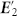 (Fig. 1(b1)), we can get an inverted Y-type system, in which there will be ***E****_S_* and ***E****_F_*_2_ but no ***E****_F_*_1_. As indicated by Fig. 4(f), ***E****_S_* signal is naturally used as a seed injected into the Stokes port of SP-FWM process and amplified as 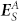. Then, the 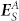 signal, mode-matched to the cavity, forms a cavity mode. In free space, the measured coexisting spectrum of 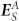 and ***E****_F_*_2_ versus δ with Δ_2_ = 1.2 GHz is displayed as the bottom curve in the inset of Fig. 4(a), in which a narrow peak labeled as “S” (corresponding to EIA dip labeled as “E” on the top curve) and a broad signal can be seen. The frequency of peak “S” changes with Δ_2_, so peak “S” is the signal 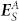, while the broad signal is ***E****_F_*_2_ without the EIA window. With both 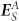 and ***E****_F_*_2_ resonant in the cavity, the measured CTS versus δ is given by the bottom solid curve in Fig. 4(a), where we can see five peaks labeled as “P1”, “P2”, “ST” “P3”, and “P4”, respectively, and the peak “ST” corresponds to the EIA dip “E” on the top dash-dotted curve. Without ***E***_2_, both the “ST” peak and “E” dip disappear in Fig. 4(b). So we can identify the “ST” peak is the transmission peak of 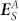, and the other four peaks are the cavity modes of ***E****_F_*_2_ picked out by the cavity windows from the broad gain. Tuning the cavity length to make a cavity transmission peak of ***E****_F_*_2_ overlap with the “ST” peak (i.e. the cavity window overlaps with the EIA window), the resulted CTS (the solid curve) and the corresponding EIA (the dash-dotted curve) versus δ are shown in Fig. 4(c), where the coexisting cavity modes of the SWM (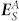) and FWM (***E****_F_*_2_) signals are obtained. By blocking ***E***_2_ at this time, the cavity mode of the SWM signal disappears, and only the cavity modes of ***E****_F_*_2_ survive (dashed line of Fig. 4(c)). Comparing the results with and without ***E***_2_ in Fig. 4(c), the enhancement (bright-state polariton of 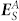) due to the coupling of cavity mode of 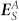 with atoms can be seen. Also, the pulling of the cavity resonant frequency due to the dispersion change induced by ***E***_2_^20^,^21^ can be observed. By fixing Δ_1_ and Δ_2_ at the point of coexisting cavity modes and scanning Δ*_ac_* in Fig. 4(d), the measured coexisting cavity modes of 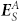 and ***E****_F_*_2_ versus Δ*_ac_* are shown on the solid curve, while the dashed curve shows the cavity modes of ***E****_F_*_2_ without ***E***_2_. Also, the pulling of the cavity resonant frequency is observed in Fig. 4(d). According to the coupled atom-cavity model, the CTS amplitude of 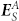 is given by 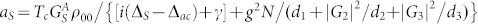, where Δ*_S_* = Ω_1_ − *ω_S_*, *d*_3_ = Γ_30_ + *i*(Δ_1_ − Δ_3_), and 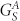 is the Rabi frequency of 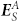. The transmission coefficient of intensity of ***E****_F_*_2_ is 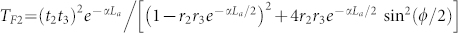, where *r_i_* (*t_i_*) is the reflection (transmission) coefficient of the mirror *M_i_* of the cavity with 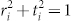, and *ϕ*(*ω_F_*_2_) = 2*π*(Δ*_ac_* − Δ_1_)/*ω_FSRE_* + (*n* − 1)*L_a_ω_F_*_2_/*c* is the round-trip phase shift experienced by ***E****_F_*_2_, with light speed in vacuum *c*, length of the atomic cell *L_a_*, and FSR of the empty optical cavity *ω_FSRE_* = *c*/*L_c_* with a cavity length *L_c_*. The terms *α* = 2(*ω_F_*_2_/*c*)Im[(1 + *χ*)^1/2^] and *n* = Re[(1 + *χ*)^1/2^] are the intensity absorption coefficient and refractive index of the atomic medium, respectively, with *χ* = *i*2*g*^2^*NL_c_*/[*L_a_ω_F_*_2_(*d*_1_ + |*G*_2_|^2^/*d*_2_ + |*G*_3_|^2^/*d*_3_)] when only the linear susceptibility is considered. Using the expressions of *a*_S_ and *T_F_*_2_, the calculated normalized CTS of coexisting 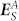 and ***E****_F_*_2_ versus δ are shown on the bottom curve in Fig. 4(e), which agrees with the experimental result.

Besides, the EIA peaks induced by the multiple cavity modes of ***E****_F_*_2_ are observed in Fig. 4(b), where each inset is a measured EIA peak multiplied by 10. Because each cavity mode of ***E****_F_*_2_ couples to |0〉→|1〉 and propagates along the same direction as ***E***_1_, the two-photon resonance (one photon from ***E***_1_ and another from ***E****_F_*_2_) induces the Doppler-free atomic coherence^23^. Owing to the far detuning of the cavity modes of ***E****_F_*_2_, the EIA is induced instead of EIT. The probe absorption can be obtained by 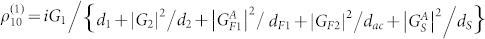 with *d_F_*_1_ = *i*(Δ_1_ − Δ*_F_*_1_) + Γ_10_, *d_ac_* = *i*(Δ_1_ − Δ*_ac_*) + Γ_10_ and *d_S_* = *i*(Δ_1_ − Δ*_S_*) + Γ_10_, which indicates ***E***_2_ field and the cavity modes of 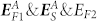 with far detuning can all induce EIA. However, the induced EIA by the cavity modes of 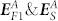 are not easy to be observed separately, because they overlap with the EIA created by the ***E***_2_ field, which is much stronger than the cavity modes. The induced EIA (insets of Fig. 4(b)) by the cavity modes of ***E****_F_*_2_ can be observed by tuning Δ*_ac_* or Δ_2_ to separate it from the EIA of ***E***_2_. The calculated probe absorption by 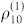 is shown on the top curve in Fig. 4(e), where the highest EIA peak is the sum from ***E***_2_ and cavity modes of 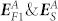, and the other peaks are induced by the cavity modes of ***E****_F_*_2_.

## Discussion

We have experimentally studied the bright-state polaritons of multi-wave mixing signals through OPA processes in a multi-level atom-cavity composite system. It is demonstrated that the polaritons are much narrowed due to EIA and cavity window. It would be important for the narrow-band long-distance quantum communications. Besides, coexisting cavity modes of the generated SWM and FWM signals are also observed, which can help us to better understand the interactions between the strongly coupled multi-level atoms and the optical cavity. Moreover, the induced EIA by the multiple FWM cavity modes in the probe spectrum is observed. Such investigation in an atom-cavity coupling system may find potential applications in building multi-channel nonlinear optical devices for quantum information processing.

## Methods

### Experiment setup

The atom-cavity composite system is shown in Fig. 1(a), where a 7 cm long Rb atomic cell is placed inside a 38 cm long optical ring cavity. The concave mirrors M1 and M2 (with the same radius of curvature of 100 mm) have 99.9% and 97.5% reflectivities at 780 nm, respectively. The flat mirror M3 with a reflectivity of 97.5% at 780 nm is used as the output coupler. M1 is mounted on a piezoelectric transducer (PZT) to adjust and lock the cavity length. The atoms can be viewed as an inverted four-level Y-type system as shown in Fig. 1(b1), where the relevant energy levels are 5*S*_1/2_(*F* = 3)(|0〉), 5*P*_3/2_(|1〉), 5*D*_3/2_(|2〉) and 5*S*_1/2_(*F* = 2)(|3〉) in ^85^Rb. Three grating-stabilized diode lasers are used as the probe field ***E***_1_ (frequency *ω*_1_, wave vector ***k***_1_, Rabi frequency *G*_1_, and wavelength 780.2 nm), pumping fields ***E***_2_ (*ω*_2_, ***k***_2_, *G*_2_, and 776.16 nm) & 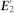 (*ω*_2_, 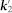, 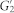), and coupling fields ***E***_3_ (*ω*_3_, ***k***_3_, *G*_3_, and 780.2 nm) & 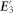 (*ω*_3_, 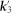, 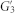), where ***E***_1_ drives the transition |0〉→|1〉, ***E***_2_ & 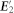 drive |1〉→|2〉, and ***E***_3_ & 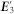 drive |1〉→|3〉. The ***E***_2_ (with horizontal polarization) and 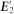 (with vertical polarization) beams come from the same laser. 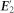 beam propagates along the optical axis of the cavity, and ***E***_2_ beam co-propagates with 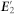 beam having a 2° angle between them. The ***E***_1_ (vertically polarized) beam counter-propagates with ***E***_2_ beam; that is the two-photon Doppler-free scheme for ladder-type system^23^. The CTS and absorption of ***E***_1_ is detected by avalanche photodiode detector 1 (APD1) and APD2, respectively. The ***E***_3_ and 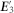 beams have the same propagation directions and polarizations with ***E***_2_ and 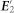 beams, respectively.

### Theoretical model

According to the perturbation chains^15^, the density-matrix elements for ***E****_F_*_1_ and ***E****_S_* are 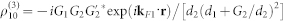 and 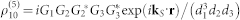, where *d*_1_ = Γ_10_ + *i*Δ_1_, *d*_2_ = Γ_20_ + *i*(Δ_1_ + Δ_2_) and *d*_3_ = Γ_30_ + *i*(Δ_1_ − Δ_3_) with the decay rate Γ*_ij_* between states |*i*〉 and |*j*〉. The generated ***E****_F_*_1_ (or ***E****_S_*) signal is injected into the Stokes port of the SP-FWM process, then parametrically amplified and denoted as 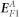 (or 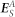). The photon numbers of the output Stokes (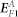 or 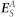) and anti-Stokes fields of the OPA are 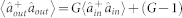 and 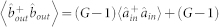, where 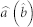 is the annihilation operator of ***E****_St_* (***E****_ASt_*), and 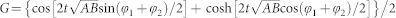 is the gain of the process with the modules *A* and *B* (phases *φ*_1_ and *φ*_2_) defined in 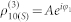 and 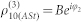 for ***E****_St_* and ***E****_ASt_*, respectively^18^.

Then, we describe the theoretical model of such coupled atom-cavity system. Here, we just discuss the model with the cavity mode of 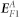 coupling with atoms, since the case for 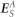 can be discussed in the same way. In the limit of weak cavity field and with all the atoms initially in the ground state |0〉, the system can be described by the equations 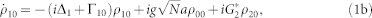

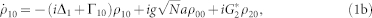

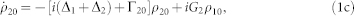
where *a* is the amplitude of the cavity field 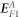, *ρ_ij_* the density-matrix element, *γ* the cavity loss, *g* the single-atom-cavity coupling strength, *N* the number of atoms in the cavity, 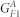 the Rabi frequency of 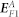, Δ*_F_*_1_ = Ω_1_ − *ω_F_*_1_, and cavity frequency detuning Δ*_ac_* = Ω_1_ − *ω_c_* (with *ω_c_* being the resonant frequency of the cavity). So, the amplitude of CTS is given by 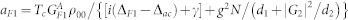, where *T_c_* is the transmission function without dressing effect. The frequency *ω_F_*_1_ of the FWM signal is identical with *ω*_1_ to obey the energy conservation, giving Δ*_F_*_1_ = Δ_1_. According to the same coupled atom-cavity model, the CTS amplitude of 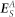 is given by 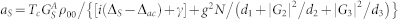.

## Author Contributions

H.C. and Y.Z. wrote the main manuscript text and contributed to the theoretical and experimental analysis of this work. X.Y., Z.W. and X.Z. prepared figures 1–4. Y.Z. and M.X. provided the idea. All authors reviewed the manuscript.
